# Health Literacy, Health Care Utilization, and Direct Cost of Care Among Linguistically Diverse Patients with Type 2 Diabetes Mellitus

**DOI:** 10.3928/24748307-20170613-01

**Published:** 2017-07-31

**Authors:** Gerardine Doyle, Sarah Gibney, Judy Quan, Ulrik Martensen, Dean Schillinger

## Abstract

**Background:**

Low functional health literacy (HL) has been associated with poor self-management of chronic conditions, including type 2 diabetes mellitus (T2DM), an inefficient use of health services, and higher health care costs. Low functional HL and limited English language proficiency both independently predict poor glycemic control among Latino and Chinese immigrants in the United States, and is more common among patients with diabetes with limited HL.

**Objective:**

This study investigated the relationship between low functional HL, health care utilization, and costs of health care among a cohort of low-income patients with T2DM whose primary language was English, Spanish, or Cantonese (N = 277).

**Methods:**

Patient data were collected from Medicaid administrative health care records as part of a low-income managed care program administered by the San Francisco Health Plan between April 2009 and March 2011. HL was measured with the Brief Questions Screening Tool for Health Literacy, administered via telephone survey. We used negative binomial regression with robust standard errors to estimate the effect of low functional HL on health care utilization, adjusting for demographic, socioeconomic, and health covariates. Results were reported as rate-ratios (RRs). We used two-part regression models to estimate the marginal difference in cost per patient associated with low functional HL. Utilization and cost models were also estimated, stratified by patient language.

**Key Results:**

We observed a nonsignificant association between low functional HL and lower health care utilization, and lower total health care costs (−$1,493.53, 95% confidence interval [CI]: $3,602 to $615). While we observed a nonsignificant trend for low functional HL and lower utilization and total cost among people who speak English and Cantonese, low functional HL was significantly associated with more outpatient visits among patients who spoke Spanish (RR 1.31, 95% CI 1 to 1.72).

**Conclusions:**

The relationship between low functional HL and health care utilization among this linguistically diverse cohort of patients with T2DM varied by patient language. Further research is needed to determine if lower utilization and costs in certain linguistic subgroups is indicative of barriers to access. **[*Health Literacy Research and Practice*. 2017;1(3):e116–e126.]**

**Plain Language Summary:**

This study attempts to understand the relationship between health literacy, health care utilization, and costs of health care among an ethnically and linguistically diverse cohort of low-income patients with type 2 diabetes mellitus. We observed differences that could be due to actual differential effects of low HL by language status, or could be explained by unmeasured differences in health-seeking behaviors, access to care, degree of acculturation, or comorbidities.

Global population aging, changing nutritional environments, and low physical activity levels are placing increasing numbers of people at risk for being diagnosed with type 2 diabetes mellitus (T2DM) as well as intensifying the cost burden of diabetes for patients and health systems (International Diabetes Federation, 2013). Recent estimates from the International Diabetes Federation indicate that at least $144.3 billion was spent on diabetes health care in the European Region, and that at least $310 billion was spent in North America and the Caribbean in 2014 (International Diabetes Federation, 2014). Diabetes is associated with increased mortality and morbidity, including retinopathy, renal disease, lower extremity amputations, cardiovascular diseases, alongside increased usage of both the health care system and medication (Zhang, Zhang, Brown et al., 2010). Cost control and reduction may be achieved with successful disease management that prevents complications, controls comorbidities, and reduces inefficient health care utilization (Guo, Gibson, Gropper, et al., 1998).

Health literacy (HL) describes the functional, interactive, and critical skills needed to optimize health and reflect a person's capacity to function optimally, as an engaged patient, in the health care system (Nutbeam, 2000). Low functional HL has been linked to suboptimal health behavior, poorer health outcomes, and poorer self-management of chronic conditions, including T2DM (Levin-Zamir & Peterburg, 2001). Low functional HL has been associated with increased hospitalization (Berkman, Sheridan, Donahue, Halpern, & Crotty, 2011) and increased cost of care across patients with different conditions (Franzen, Mantwill, Rapold, & Schulz, 2013; Eichler, Wieser, & Brügger, 2009; Howard, Gazmararian & Parker, 2005). Among patients with diabetes, low functional HL was associated with higher outpatient costs and frequency of physician visits (Franzen et al., 2013). The annual cost of HL to the health care system has been estimated to range from 3% to 5% of the total health care budget (Eichler, Wieser, & Brügger, 2009), and patients with low functional HL use an “inefficient” mix of health care services and generate higher emergency department costs (Howard et al., 2005).

In addition to using the health systems more frequently and incurring higher health care costs, low functional HL has been associated with poorer self-management of chronic conditions (Levin-Zamir & Peterburg, 2001; Kickbusch, Pelikan, Apfel, et al., 2013). Diabetes requires intensive self-management and HL is considered a key determinant of successful self-management and treatment outcomes (Cavanaugh, 2011; Kim, Love, Quistberg, Shea, 2004; Schillinger et al., 2002). Studies have found that patients with low functional HL receive substandard health care access, resulting in poorer quality of care (Schillinger & Chen, 2004). In addition to other socio-economic and lifestyle factors, low functional HL and limited English proficiency have been found to independently predict poor glycemic control among Latino and Chinese immigrants in the United States (Chesla, Chun, & Kwan, 2009; Fernandez et al., 2010; Sudore et al., 2009). Cultural beliefs about health play an important role in diabetes self-management (Chesla, et al., 2009; Nam, Chesla, Stotts, Kroon & & Janson, 2011) and communication barriers may play an important role in clinicians' inability to effectively promote TDM2 self-management skills. Although prior studies have identified an association between low functional HL, poor self-management of chronic illness, and cost of care, the findings are inconsistent. Our study seeks to address these inconsistent findings by addressing a gap in the literature, first to understand the role that health service utilization may play in explaining some of this association, and second to determine how cultural beliefs may also affect access. Although low HL has been associated with poor glycemic control and higher rates of complications in people with T2DM cared for in safety net settings (Schillinger et al., 2002), to our knowledge, no previous study has examined the relationship between HL and diabetes-related health care utilization or costs. We seek to better understand the association between health service utilization, health outcomes for T2DM, and HL.

The European Commission's FP7 Diabetes Literacy Consortium conducted a program of research on the cost-effectiveness of diabetes self-management (Van den Broucke et al., 2014), and although empirical investigations of HL and costs in the US and Europe have increasingly relied on administrative data sources that capture direct cost of care, health care utilization, and HL status (Baker et al., 2002; Pletscher, 2013; Weiss & Palmer, 2004; Wieser, Moschetti, Eichler, Holly & Brügger, 2008), few of these studies have focused exclusively on patients with T2DM.

There is a dearth of literature on the treatment costs of T2DM and so such costs are not fully understood. In the US, previous studies have estimated that inpatient care accounted for 43% of total T2DM health care expenditure, followed by medications for treatment and prevention of diabetic complications (18%) (Yang, et al., 2013). Furthermore, hospitalization has been estimated to account for 29.9% of total direct cost-of-illness (Guo, et al., 1998). The number of comorbidities, frequency of physician visits, insulin dependence, and diabetic complications account for much of the direct costs of illness among Medicaid patients with diabetes (Guo, Gibson, Gropper, et al., 1998). Recent studies (Dall et al., 2014, 2010) have estimated the economic burden associated with diagnosed and undiagnosed diabetes, gestational, and pre-diabetes in adults. Dall et al., (2014) found this to be $322 billion in 2012. Yet, despite its prevalence and the cost burden implied in that prevalence, estimates of these costs vary widely. It is our opinion that to inform policy, a far more careful analysis is necessary, whereby bottom-up patient level cost information is derived on the cost of care of T2DM and its associated complications rather than top-down economic estimates of the cost burden. The prevalence of low functional HL among patients with T2DM has been estimated at between 25% and 40% (Franzen, et al., 2013), and research in this area is increasing.

In high and middle-income countries, ethnic minority groups often have a higher prevalence of diabetes than other population groups (Attridge, Creamer, Ramsden, Cannings-John, & Hawthorne, et al., 2014), and migrant groups can experience low-quality access to health care and health promotion services (HLS-EU Consortium, 2012). Language and cultural traditions may act as a barrier (Chesla, 2009; Anderson, Scrimshaw, Fullilove, Fielding & Normand, 2003). Limited English proficiency has been shown to independently predict for poor glycemic control among Latin Americans with diabetes, and is more common among patients with limited HL (Fernandez, et al., 2011). When care is provided by physicians who do not speak a patient's native language, that barrier may exacerbate and even supersede the negative effect of low functional HL on patient-physician communication (Fernandez, Schillinger, Warton, et al., 2011; Sudore, et al., 2009).

The current study investigated the relationship between HL, health care utilization, and direct costs of health care among an ethnically and linguistically diverse sample of low-income Medicaid-managed care patients in San Francisco, CA, who participated in a diabetes self-management intervention through their local public delivery health care system (Ratanawongsa, et al., 2012). The relationship between HL and language proficiency is complex; therefore, we also examined within each language group: English, Spanish, and Cantonese. We measured self-reported ability to read and understand basic health-related materials in health care settings that has previously been highly predictive of T2DM outcomes, including hypoglycemia, and use of a T2DM patient portal (Sarkar, Schillinger, López, & Sudore, 2010).

## Methods

### Data and Sample

Data were obtained from Medicaid administrative health care records and a low-income managed care program administered by the San Francisco Health Plan (SFHP). Survey data were obtained from an evaluation (wait-list versus control) of a 6-month T2DM self-management support program, referred to as “the parent study” hereafter (Ratanawongsa, et al., 2012). As the data for this study were collected from secondary data sources, the research team did not have any communication with the patient cohorts. Ethical approval had been obtained for the parent study from the Committee on Human Research at the University of California, San Francisco.

Medicaid serves populations who have low socioeconomic status (SES) and chronic illness (Ratanawongsa, et al., 2012). The SFHP is a nonprofit managed care plan serving a linguistically diverse, low-income Medicaid population, and a low-income and uninsured population of the city and county of San Francisco.

The current study sample comprises 277 adult members of the SFHP with T2DM. The participants included adults age 18 years or older, who spoke English, Cantonese, or Spanish and had visited 1 of 4 public clinics in San Francisco's safety net health care system within a 24-month period between April 2009 and March 2011. Participants with Medicare insurance (those older than age 64 years) were excluded from the cost analyses only and not the utilization analyses as these participants had alternative (non-SFHP) pharmacy benefits for which there was no cost information. Full details of the parent study have been described elsewhere (Ratanawongsa, et al., 2012).

### Measures

The current study used the three-item Brief Questions Screening Tool for Health Literacy that has been validated for English and Spanish speakers, but not for Cantonese speakers (Sarkar, et al., 2011; Chew, Griffin, Partin, et al., 2008). This HL measure includes three questions to detect low functional HL:

1. Problems reading: “How often do you have problems learning about your medical condition because of difficulty understanding written information?”
2. Confident with forms: “How confident are you filling out forms by yourself?”
3. Help read: “How often do you have someone like a family member, friend, hospital, or clinic worker or caregiver, help you read clinic or hospital materials?”

Responses were scored on a Likert scale from 0 to 4. “Confident with Forms” was answered with “extremely,” “quite a bit,” “somewhat,” “a little bit,” and “not at all” confident. “Problems Reading” was answered with “all of the time,” “most of the time,” “some of the time,” “a little of the time,” or “none of the time.” “Help read” was answered with “never,” “occasionally,” “sometimes,” “often,” and “always.” Total scores are divided into high HL (scores of 3–8) or low HL (scores of 9–15). The internal consistency of the summative score was sufficient: α = 0.76 (Parker, Baker, Williams, & Nurss, 1995). High HL scores (<9) were used as the reference category in all analyses.

The health care utilization measures examined were count-based and included days spent in hospital, number of hospitalizations, number of emergency department visits, and number of outpatient visits. Health care costs for each medical encounter were estimated based on a fee schedule maintained by the SFHP managed care plan, where rates are negotiated across health systems. These costs included ambulance, emergency department, hospitalization, outpatient, and pharmacy.

The baseline socio-demographic characteristics of the cohort included age, gender (male/female), and self-identified primary language (English, Spanish, Cantonese). Socioeconomic characteristics included insurance types: Medicare, Medicaid, Healthy Workers/Healthy San Francisco Health Plan, and other (uninsured and commercial). Baseline health status was self-reported and included as a binary variable in analysis: excellent, very good, good, or fair/poor. Diabetes duration (in years) at baseline was captured by the question “How many years have you had diabetes?” Insulin use at baseline was self-reported (yes or no), based on the question “Have you been prescribed or asked by a member of your diabetes healthcare team to take insulin?” Patients who had been prescribed insulin but were not currently taking it were categorized as “no.”

### Data Analysis

We compared baseline socio-demographic and health-related characteristics of patients with low vs. high HL by using chi-square or Fisher's Exact tests for categorical variables, *t*-tests for interval variables if normally distributed, and Wilcoxon rank sums tests for interval variables not normally distributed. We used negative binomial regression analysis with robust standard errors to estimate the effect of low (versus high) HL on health care utilization. Models were adjusted for language, age, treatment group (according to the parent-study), diabetes duration, health insurance status, insulin use, and self-rated health. Results are reported as rate ratios (RRs), with corresponding 95% confidence intervals (CI) that represent changes in health care utilization between baseline and follow up between the groups with low and high HL levels. For interpretation, a RR of 1 indicates no difference in health care utilization between patients with low and high HL.

Following previous approaches, we used a two-part model to estimate the marginal effect (in health care costs) of having low functional HL (Duan, Manning, Morris, & Newhouse, 1983). We then repeated this analysis, stratified by language, to estimate the effect of low functional HL on health care cost by language group. These stratified models were unadjusted. Standard regression methods yield inaccurate cost estimates due to the highly skewed distribution and presence of many “zero” values. The two-part model mimics the empirical distribution of medical spending by splitting the distribution into two parts and allowing the impact of independent variables (e.g., HL) on the probability of using medical care to be independent of their impact on the costs of medical care for those who use it (Howard, 2005). The first stage of the two-part model is a logistic regression that is estimated on the entire sample. In this stage the dependent variable equals 1 if the patient used care and 0 if otherwise. The second part is a nonlinear regression estimated on only those patients who used care. This second component posits that medical expenditures equal the exponentiated sum of a linear combination of coefficients and covariates. The marginal effects should be interpreted as the instantaneous rate of change in US costs produced by a change in HL level, holding all other covariates constant.

We repeated all analysis, stratified by language group, to investigate if the relationships we observed masked differences within the groups. These stratified models were exploratory and unadjusted.

## Results

The characteristics of the sample are summarized in **Table 1**. The unadjusted and adjusted RRs of health care utilization for the 24-month follow-up period after baseline randomization, as a function of functional HL status, (low functional HL vs. high HL, N = 277) are presented in **Table 2**.

Although low functional HL was also associated with lower rates of hospitalization (number of visits) and with lower rates of emergency department (number of visits), none of these remained statistically significant in the adjusted model (Model 2). **Table 3** shows the results of three separate unadjusted models of health care utilization, stratified by primary language.

The relationship between HL and utilization varied by language group. Among English speakers, we found no significant differences by HL status in any of the health care utilization domains measured. Among Spanish speakers, we observed a significantly higher RR for outpatient use associated with low functional HL (RR 1.31; 95% CI: 1 to 1.72) and a nonsignificant trend of more frequent health care utilization for all other domains measured. Among Cantonese speakers, the opposite pattern was observed: a lower RR among the low functional HL group across all health care utilization domains. However, these results were not significant.

**Table 4** shows the marginal effect on costs in US dollars associated with low functional HL. In the adjusted model, low functional HL was associated with a mean nonsignificant reduction in total health care costs of −$1,493.54 per participant over the 2-year period (95% CI: −$3,602 to $615; *p* = .17) and lower outpatient costs per participant (−$1,098; 95% CI: −$2,338 to $141; *p* = .08).

**Table 5** illustrates the unadjusted marginal health care costs by HL for each language group. We observed only one statistically significant relationship in stratified analyses: there were higher pharmacy costs among low functional HL Cantonese speakers compared to high HL Cantonese speakers ($561; 95% CI: $12 to $1,110). Among English speakers, low functional HL did not appear to be associated with higher health care costs; among Spanish speakers, low functional HL appeared to be associated with higher health costs; and among Cantonese speakers, except for pharmacy costs, low functional HL appeared to be associated with lower health costs.

## Discussion

Using data from the SFHP, this study examined health care utilization and costs among ethnically diverse patients with diabetes across low and high HL levels. We observed several nonsignificant trends and differences in health care utilization between the low and HL group. These associations were consistent with a previous study of HL and health care utilization among older Medicare enrollees (Cho, Lee, Arozullah, & Crittenden, 2008), which showed a weak relationship between low functional HL levels and hospital admissions. For some ethnic groups, our findings contrast with those (Baker, et al., 2002) who demonstrated that low functional HL predicted a higher rate of hospital admissions among Medicare enrollees. Similarly, Howard et al., (2005) found that older patients with low functional HL showed an increased risk of hospitalization as well as an inefficient use of a mix of services, when controlled for age, gender, race/ethnicity, income, education, tobacco and alcohol consumption, and self-reported comorbid conditions. Our findings are consistent with previous studies that focused on the general population. However, studies that focus on older populations have found a stronger relationship between health literacy and health care utilization. Less use of health care services among patients with low functional HL within this study could be explained by the fact that an adequate HL level is an important skill needed to navigate increasingly complex health systems (Nutbeam, 2000; Kickbusch, Wait, & Magg, 2006). Cultural norms and language can act as a barrier to accessing health care and reduce the quality of the communication between patient and provider (Fernandez, et al., 2010; Sudore, et al., 2009; Chesla, Chun & Kwan, 2009; Kreps, & Sparks, 2008). Health communication should ideally be tailored to the cultural health beliefs, values, norms, and expectations of each patient (Fernandez, 2010; Kreps & Sparks, 2008).

Among Spanish speakers, low functional HL was associated with higher inpatient and outpatient care. The opposite relationship was found among Cantonese speakers. Among the English speakers, low functional HL was associated with use of fewer health care services. Although these results were nonsignificant, these findings suggest that the association between HL and health care utilization in the US may differ depending on primary language and ethnic differences in health-seeking behavior and beliefs.

When we compared the summative scores of HL, we found no statistically significant difference in health care costs. Our findings were not consistent with a previous study (Haun et al., 2015), which found higher costs of care across an entire cohort (now just diabetics) and also found that T2DM was more prevalent among those with low HL. This association was partially explained by the organization of the health care system that ensured fast and easy access to physicians in private practices (Eichler, et al., 2009). Our results were also inconsistent with studies from the US showing that lower HL was associated with higher emergency department and in-patient costs (Howard, et al., 2005).

Among the different language groups, Spanish speakers with low functional HL incurred higher costs compared to those with high HL. The unadjusted analyses could indicate that there may be language-barriers to seeking out appropriate health care services for patients in the Spanish and Cantonese speaking group. In general, our results indicate that patients in the low functional HL group incur lower costs to the health care system, which is likely to be linked to less frequent use of in-patient services. It is unclear from this analysis whether this is due to a barrier, reduced need, or cultural and ethnic difference in health-seeking behaviors.

### Study Limitations

This study has several study limitations that should be considered. To begin, the sample consisted of 278 adults who were part of the SFHP. These patients were mostly (80%) born outside of the US. Therefore, our results may have limited external validity. Second, we may not have adjusted for all variables that predict health care utilization. Health insurance status was used as a measure of SES; however, this variable may not accurately capture other aspects of SES such as education. Although we controlled for health status, we did not control for comorbidities. At least 65% of adults age 65 years and older live with two or more chronic conditions, and among diabetes patients 40% live with at least three (Wolff, Starfield, & Anderson, 2002). Third, our results are based on three questions from the Short Test of Functional Health Literacy in Adults, a functional health literacy measure that captures a person's ability to read and understand health-related materials in health care settings and a patient's ability to apply literacy skills to materials such as prescriptions and medicine labels (Parker, Baker, Williams, et al., 1995; Baker, Williams, Parker, Gazamararian & Nurss, 1999). This measure does not capture the empowerment dimensions of HL, which enable people to self-manage their health across the life course (Nutbeam, 2000). The advantage of using these screening questions is that they effectively detect low functional HL and are accessible and easy to understand (Chew, et al., 2008). However, these measures were developed and validated for English-speaking populations and, therefore, have not been validated among Cantonese speakers to date. Further validation of these measures among different language groups is required to support future research among linguistically diverse patient populations. Although our study examined the primary language of the participants, the language of their providers and/or use of medical interpreters during office visits may also be associated with the frequency of visits. However, we did not have measures of provider language or use of interpreters within our dataset. Previous studies have demonstrated discrepancies between patients' and doctors' impressions of patient knowledge (Olson & Windish, 2010; Janz, et al., 2004). In terms of doctor-patient interactions and concordance, observational studies are needed to understand how health professionals interact with and assess their patients. Finally, medical spending data are highly skewed (Wolff, et al., 2002). This means that a group of patients may not have medical expenses at all, whereas others do. In addition, those who use the health care system may contribute substantially more than the “average” consumer of health care, especially for patients with comorbidities. This could result in an uneven distribution of health care spending. Even though we used a two-part model to account for the data being skewed, Howard et al. (2005) argue that large sample sizes are needed to precisely identify differences between spending across groups. Therefore, in the interpretation of our results, it is important to be cognizant of our relatively small sample size of 277 participants. Although our study investigated the relationship between low functional HL, health care utilization, and costs among a cohort of low-income T2DM patients whose primary language was English, Spanish, or Cantonese, our study did not encompass any cultural barriers to accessing, understanding, and applying health information. This is an important area in which there is a significant gap in the extant literature, and requires future research.

In this ethnically and linguistically diverse cohort, we found relationships between HL, utilization, and costs that appeared to vary by primary language status. These observed differences could be due to actual differential effects of low HL by language status, or could be explained by unmeasured differences in health-seeking behaviors, access to care, degree of acculturation, or comorbidities.

## Figures and Tables

**Table 1:** Sample Characteristics of Adults with Type 2 Diabetes Mellitus Enrolled in the San Francisco Health Plan

**Sample Characteristics**	**All Patients (*n* = 277)**	**High HL (*n* = 159)**	**Low HL (*n* = 118)**	***p* Value**

Mean age in years (*SD*)	55.81 (8.26)	54.63 (9.24)	57.40 (6.43)	.009^a^

Age range (years)	18–75	18–74	39–75	

Gender				
Male	70 (25.3%)	45 (28.3%)	25 (21.2%)	.18
Female	207 (74.7%)	114 (71.7%)	93 (78.8%)	

Ethnic group				
Hispanic or Latino	69 (24.9%)	41 (25.8%)	28 (23.7%)	.7
Non-Hispanic or Non-Latino	208 (75.1%)	118 (74.2%)	90 (76.3%)	

Race				
Asian/Pacific				
Islander	169 (61%)	83 (52.2%)	86 (72.9%)	< .0001^b^
Black	20 (7.2%)	19 (11.9%)	1 (0.8%)	
Hispanic	63 (22.7%)	36 (22.6%)	27 (22.9%)	
Native American/multiethnic/other	6 (2.2%)	4 (2.5%)	2 (1.7%)	
White	19 (6.9%)	17 (10.7%)	2 (1.7%)	

Language				
English	75 (27.1%)	66 (41.5%)	9 (7.6%)	< .0001
Cantonese	149 (53.8%)	66 (41.5%)	83 (70.3%)	
Spanish	53 (19.1%)	27 (17%)	26 (22%)	

Insurance type				
Medi-Cal	57 (20.7%)	36 (22.6%)	21 (17.9%)	.03^b^
Medicare	12 (4.3%)	11 (6.9%)	1 (0.9%)	
Commercial/uninsured	3 (1.1%)	1 (0.6%)	2 (1.7%)	
Healthy worker/San Francisco Health Plan	204 (73.9%)	111 (69.8%)	93 (79.5%)	

Mean diabetes duration (years) (*SD*)	7.03 (5.68)	7.22 (5.78)	6.78 (5.55)	.63^a^

Using insulin				
No	227 (81.9%)	130 (81.8%)	97 (82.2%)	.92
Yes	50 (18.1%)	29 (18.2%)	21 (17.8%)	

Self-rated health				
Excellent/very good/good	101 (36.5%)	74 (46.5%)	27 (22.9%)	--
Fair/poor	176 (63.5%)	85 (53.5%)	91 (77.1%)	

Treatment group				
Intervention	144 (52%)	78 (49.1%)	66 (55.9%)	.26
Waitlist	133 (48%)	81 (50.9%)	52 (44.1%)	

Note. Low HL is defined as a score of 9 or higher (out of 15) for the summative score of all three health literacy items. HL = health literacy; SD = standard deviation.

a *p* Values derived from chi-square or Fisher's Exact tests for categorical variables.

b *t* tests for interval variables if normally distributed and Wilcoxon rank sums tests if interval variables not normally distributed.

**Table 2:** Unadjusted and Adjusted Rate Ratios of Health Care Utilization by Health Literacy (Low vs. High HL)

**Variable**	**Model 1 (Unadjusted)**				**Model 2 (Adjusted)**			
	**RR**	***p***	**CI (95%)**		**RR**	***p***	**CI (95%)**	
			**Lower bound**	**Upper bound**			**Lower bound**	**Upper bound**
Hospitalizations (number of visits)	0.4	.04	0.17	0.95	0.66	.42	0.25	1.79
Days in hospital)	0.41	.09	0.15	1.15	0.63	.36	0.23	1.7
Emergency department (number of visits)	0.48	.04	0.25	0.96	0.86	.6	0.48	1.52
Emergency department and hospitalizations	0.44	.02	0.23	0.87	0.76	.34	0.43	1.34
Outpatient visits	0.9	.17	0.78	1.05	0.99	.83	0.87	1.12

Note. N = 277. Data represent a 24-month follow-up period. Binary categories of HL summative score were 9–15 (low HL) vs. 3–8 (high HL). CI = confidence interval; HL = health literacy; RR = rate ratios.

**Table 3:** Unadjusted Rate Ratios of Health Care Utilization by Health Literacy, Stratified by Primary Language

**Variables**	**English (*n* = 85)**				**Spanish (*n* = 48)**				**Cantonese (*n* = 144)**			
	**RR**	***p***	**CI (95%)**		**RR**	***p***	**CI (95%)**		**RR**	***p***	**CI (95%)**	
			**Lower bound**	**Upper bound**			**Lower bound**	**Upper bound**			**Lower bound**	**Upper bound**
Hospitalizations (number of visits)	0.96	.95	0.28	3.27	1.85	.49	0.32	10.60	0.22	.07	0.04	1.11
Days in hospital)	0.61	.51	0.14	2.67	1.64	.64	0.21	12.64	0.34	.22	0.06	1.87
Emergency department (number of visits)	0.76	.65	0.23	2.46	2.44	.06	0.96	6.18	0.63	.33	0.25	1.60
Emergency department and hospitalizations	0.68	.51	0.21	2.19	2.31	.06	0.96	5.54	0.56	.20	0.23	1.37
Outpatient visits	0.97	.86	0.70	1.35	1.31	.05	1	1.72	0.94	.40	0.81	1.09

Note. Binary categories of health literacy summative scores were 9–15 (low HL) vs. 3–8 (high HL). Data represent a 24-month follow-up period. CI = confidence interval; HL = health literacy; RR = rate ratios.

**Table 4:** Marginal Effect of Low Health Literacy on Direct Health Care Costs

**Variable**	**Model 1 (Unadjusted-Equal Variances)**				**Model 2 (Adjusted)^a^**			
	**Point estimate ($)**	***p***	**CI (95%)**		**Point estimate ($)**	***p***	**CI (95%)**	
			**Lower bound**	**Upper bound**			**Lower bound**	**Upper bound**
Ambulance	−0.26	.59	−1.21	0.69	−0.97	.86	−11.36	9.41
Emergency department visitst	−11.1	.5	−43.47	21.26	−11.26	.75	−80.65	58.12
Hospitalizations	−24.27	.59	−113.10	64.56	8.74	.96	−301.57	319.06
Outpatient visits	−1,219.96	.16	−2,936.29	496.37	−1,098.4	.08	−2,337.82	141.02
Pharmacy uses	293.67	.39	−371.78	959.13	396.84	.39	−509.07	1,302.75
All costs	−1,919.32	.17	−4,673.78	835.13	−1,493.53	.17	−3,602.21	615.13

Note. N = 277. Data represent a 24-month follow-up period. Binary categories of HL summative score were 9–15 (low HL) vs. 3–8 (high HL). CI = confidence interval; HL = health literacy; RR = rate ratios.

a Age, language, treatment group, duration of diabetes (years), insurance status, insulin use, and self-rated health.

**Table 5:** Unadjusted Marginal Effect of Low Health Literacy on Direct Health Care Costs, Stratified by Primary Language

**Variables**	**English (*n* = 85)**				**Spanish (*n* = 48)**				**Cantonese (*n* = 144)**			
	**Point estimate ($)**	***p***	**CI (95%)**		**Point estimate ($)**	***p***	**CI (95%)**		**Point estimate ($)**	***p***	**CI (95%)**	
			**Lower bound**	**Upper bound**			**Lower bound**	**Upper bound**			**Lower bound**	**Upper bound**
Ambulance^a^	−4.06	.54	−17.11	9					0.11	.54	−0.24	0.46
Emergency department visits	15.5	.9	−238.53	269.71	−8.03	.88	−107.92	91.87	−1.28	0.63	−6.48	3.92
Hospitalizations	−557.9	.22	−1,452.98	337.18	124.26	.61	−358.48	607	22.45	.27	−17	61.90
Outpatient visits	−198.65	.96	−7,119.30	6,722	−151.12	.87	−1,933.13	1,630.89	−138.48	.76	−1,016.4	739.49
Pharmacy use^b^					528.14	.64	−1,687.75	2,744.02	560.92	.045	11.86	1,109.97
All costs	−1,248.83	.78	−9,881.67	7,384.01	3,237.72	.34	−3,379.59	9,855.03	−141.14	.82	−1,363.6	1,081.28

Note. Binary categories of health literacy summative scores were 9–15 (low HL) vs. 3–8 (high HL). Data represent a 24-month follow-up period. CI = confidence interval; HL = health literacy; RR = rate ratios.

a Noncalculable due to no cost available for 24-month period for high literacy group.

b Noncalculable due to no cost available for 24-month period for low literacy group.
